# Intracellular Chloride Channels: Novel Biomarkers in Diseases

**DOI:** 10.3389/fphys.2020.00096

**Published:** 2020-02-14

**Authors:** Shubha Gururaja Rao, Neel J. Patel, Harpreet Singh

**Affiliations:** ^1^Department of Physiology and Cell Biology, The Ohio State University Wexner Medical Center, Columbus, OH, United States; ^2^Department of Cardiology, Hospital of the University of Pennsylvania, Philadelphia, PA, United States

**Keywords:** mitochondria, chloride intracellular channel, cancer, physiology, cell signaling, autosomal recessive nonsyndromic hearing impairment, pulmonary hyperetnsion, chloride channel

## Abstract

Ion channels are integral membrane proteins present on the plasma membrane as well as intracellular membranes. In the human genome, there are more than 400 known genes encoding ion channel proteins. Ion channels are known to regulate several cellular, organellar, and physiological processes. Any mutation or disruption in their function can result in pathological disorders, both common or rare. Ion channels present on the plasma membrane are widely acknowledged for their role in various biological processes, but in recent years, several studies have pointed out the importance of ion channels located in intracellular organelles. However, ion channels located in intracellular organelles are not well-understood in the context of physiological conditions, such as the generation of cellular excitability and ionic homeostasis. Due to the lack of information regarding their molecular identity and technical limitations of studying them, intracellular organelle ion channels have thus far been overlooked as potential therapeutic targets. In this review, we focus on a novel class of intracellular organelle ion channels, Chloride Intracellular Ion Channels (CLICs), mainly documented for their role in cardiovascular, neurophysiology, and tumor biology. CLICs have a single transmembrane domain, and in cells, they exist in cytosolic as well as membranous forms. They are predominantly present in intracellular organelles and have recently been shown to be localized to cardiomyocyte mitochondria as well as exosomes. In fact, a member of this family, CLIC5, is the first mitochondrial chloride channel to be identified on the molecular level in the inner mitochondrial membrane, while another member, CLIC4, is located predominantly in the outer mitochondrial membrane. In this review, we discuss this unique class of intracellular chloride channels, their role in pathologies, such as cardiovascular, cancer, and neurodegenerative diseases, and the recent developments concerning their usage as theraputic targets.

## Introduction

Ion fluxes and cellular physiology associated with them have been established much before the discovery of the proteins involved in their transportation. It had been long thought that transmembrane ion fluxes depend on conduction pathways located within a specific group of proteins given that the lipid bilayer was characterized as impermeable to water-soluble ions. It was not until the 1980s that a convergence of state-of-the-art biophysical and molecular biological techniques allowed in-depth characterization and identification of specific proteins responsible for ion fluxes. The major breakthroughs of the coupling of this technology included the determination of the ionic basis of action potential by Hodgkin and Huxley, the discovery of Na^+^ pump by Skou, and characterization of synaptic transmission by Kuffler, Katz, Miledi, Neher, Sakmann, Stefani, and Eccles (Hodgkin and Huxley, 1945, 1952, 1990; Skou, 1957; Eccles, 1964; Katz and Miledi, 1969; Elul et al., 1970; Bezanilla and Stefani, 1994, 1998; Stefani and Bezanilla, 1998). After the 1980s, with the advent of new technologies (Sakmann and Neher, 1984; Singh et al., 2009; Rodríguez et al., 2012), ion channels and the genes encoding them were further characterized and were found to be associated with several physiological and pathological conditions (Bezanilla and Stefani, 1994, 1998; Stefani and Bezanilla, 1998; Hille et al., 1999; Jentsch et al., 2004; Gronich et al., 2010; Maffeo et al., 2012; Feske et al., 2015). Ion channels have long been targets for pharmacologic agents in several fields, including neurological and cardiovascular therapy (Hille et al., 1999). In recent years, both cation (Toro et al., 2014) and anion channels (Ponnalagu and Singh, 2016; Gururaja Rao et al., 2018) have emerged as major molecules with aberrant expression, activity, and localization in various pathological conditions such as cardiovascular dysfunction, neurological disorders, metabolic diseases, and cancers (Cuddapah and Sontheimer, 2011). The main class of anion channels associated with various pathological disorders are chloride channels.

The role and significance of ion transport in physiology and pathology, such as the onset and development of tumors, were recognized around 100 years ago (Herl, 1924). It is well known that cells, while undergoing apoptosis, present with changes in cell volume, inter-nucleosomal DNA fragmentation or division, and apoptotic body formation. The change in cell volume during apoptosis is usually caused by alterations in intracellular ionic concentration. One of the major ions involved in cell volume regulation is chloride (Cl^–^). The role of Cl^–^ in cell proliferation was characterized as early as the 1900s (Lathrop and Loeb, 1916; Herl, 1924). On incubation of tumor cells with Locke’s (in mM, NaCl 154, KCl 5.6, CaCl_2_ 2.2 and NaHCO_3_ 2.4) or Ringer’s (in mM, NaCl 123, KCl 5.0, CaCl_2_ 1.5) solution containing higher Cl^–^ levels, but not physiological levels present in normal saline solution (15 mM NaCl), cells seldom gave rise to tumors upon transplantation into rats (Lathrop and Loeb, 1916; Herl, 1924). Cl^–^flux has also been implicated in apoptosis, specifically by modulating intrinsic and not extrinsic pathways (Heimlich and Cidlowski, 2006). Although the role of Cl^–^ in cell proliferation and apoptosis had been established over a century ago, there are still no known therapeutic targets involving Cl^–^ channels or transporters for cancer treatment due to lack of understanding of the complex cellular and molecular signaling involved in this process.

Cl^–^ channels play a vital role in cellular physiology including stabilization of cell membrane potential, transepithelial transport, maintenance of intracellular pH, cell proliferation, fluid secretion and regulation of cell volume. Anionic currents are characterized and recorded from several healthy and diseased (such as cancerous) cells (Heimlich and Cidlowski, 2006). However, the lack of information on the identity of Cl^–^ channels is the major reason for them not to be presented as targets for restoring apoptosis in tumor cells or other pathological conditions. In addition, majority of the focus on Cl^–^ channels has been on channels located at the plasma membrane, and many of those proteins are transporters [such as Chloride channels (ClCs)] and not just ion channels (Duran et al., 2010; Jentsch and Pusch, 2018). Cellular ionic concentration can modulate cell volume, and it is the intracellular ionic environment, where intracellular anion/chloride channels could play an active role, that regulates apoptosis.

All the known Cl^–^ channels can be classified as members of the voltage-sensitive ClC subfamily, transmitter-gated GABAA, and glycine receptors, calcium-activated Cl^–^ channels such as TMEM16A (Crottes and Jan, 2019), high (maxi) conductance Cl^–^ channels, the cystic fibrosis transmembrane conductance regulator (CFTR), and volume-regulated channels (Ashley, 2003). Chloride intracellular channel (CLIC) proteins are the most recent Cl^–^ channels to be discovered and are classified separately from other Cl^–^ channels; there are six members in the CLIC family (CLIC1-6) (Ashley, 2003; Littler et al., 2010; Singh, 2010; Peretti et al., 2014; Ponnalagu and Singh, 2017). CLICs are not related to the ClC family and are encoded by six different genes (*clic1-6*). Though they are highly conserved from prokaryotes to eukaryotes, including higher mammals (Gururaja Rao et al., 2017), they are not yet explored for their physiological roles. CLICs, originally named as p64, were isolated from microsomal membranes from bovine kidney and trachea (Landry et al., 1989; Redhead et al., 1992). On reconstitution in planar bilayers they presented consistent Cl^–^-selective channel activity which was sensitive to R(+)-[(6,7-Dichloro-2-cyclopentyl-2,3-dihydro-2-methyl-1-oxo-1H-inden-5-yl)-oxy] acetic acid (IAA-94) (Tonini et al., 2000; Valenzuela et al., 2000; Warton et al., 2002; Littler et al., 2004; Novarino et al., 2004; Singh and Ashley, 2006, 2007; Singh et al., 2007; Milton et al., 2008; Gururaja Rao et al., 2017; Ponnalagu and Singh, 2017; Ponnalagu et al., 2019). The fact that CLICs possess a single transmembrane domain and form a functional channel, which is extensively characterized using a planar bilayer system from bacterial to mammalian proteins, indicates that the pore is a primitive structure (Ponnalagu and Singh, 2017; Gururaja Rao et al., 2018). To form a functional channel, a minimum of four subunits are required. Occasionally individual subunits of functional units assemble to form a mega Cl^–^ channel. FRET studies involving CLIC1 indicate that CLIC1 oligomers comprise of 6–8 subunits upon oxidation in the presence of the membranes (Goodchild et al., 2009, 2010, 2011).

CLICs are known to form redox and pH-sensitive ion channels in planar bilayers (Warton et al., 2002; Singh and Ashley, 2006; Milton et al., 2008; Ponnalagu and Singh, 2017). Single-channel conductance of CLIC proteins ranges from ∼6–120 pS for CLIC1, ∼1–86 pS for CLIC4 and ∼3–400 pS for CLIC5 (Singh, 2010). So far, activity of specific CLIC proteins has not been recorded in their native environment. Structure-function carried out by Prof. Mazzanti’s group indicates that neither Arg29 nor Lys37 of CLIC1 affects the ion channel selectivity, but they report small differences in the reversal potentials in the single-channel currents and the whole-cell currents of transfected HEK cells. Our structure-function work with Cys 24 of CLIC1 did implicate the residue in redox-regulation of the channel; however, the single-channel conductance was significantly reduced. Recently, CLIC-like IAA-94 sensitive channels were recorded in the inner membrane of mitochondria which presented a single-channel conductance of ∼100 pS (Ponnalagu et al., 2019). Thus far, the only CLIC proteins known to be present in mitochondrial membranes are CLIC4 and CLIC5 (Ponnalagu et al., 2016b; Ponnalagu and Singh, 2017). CLIC5 is predominantly located in the inner mitochondrial membrane; therefore, the IAA-94 channel in mitoplast could be CLIC5. Due to variability in recording conditions, redox environment, pH and membrane composition, CLICs could be present with variable conductance (Singh, 2010; Ponnalagu and Singh, 2017). Similar to single-channel conductance, the majority of the information on their selectivity is also obtained from an artificial bilayer system. Additionally, most of the CLICs do not possess high intra anionic selectivity (Singh, 2010). Therefore, to attribute specific biophysical characteristics to individual CLICs, their activity should be recorded in their native environment, possibly with null mutants as controls.

**FIGURE 1:**
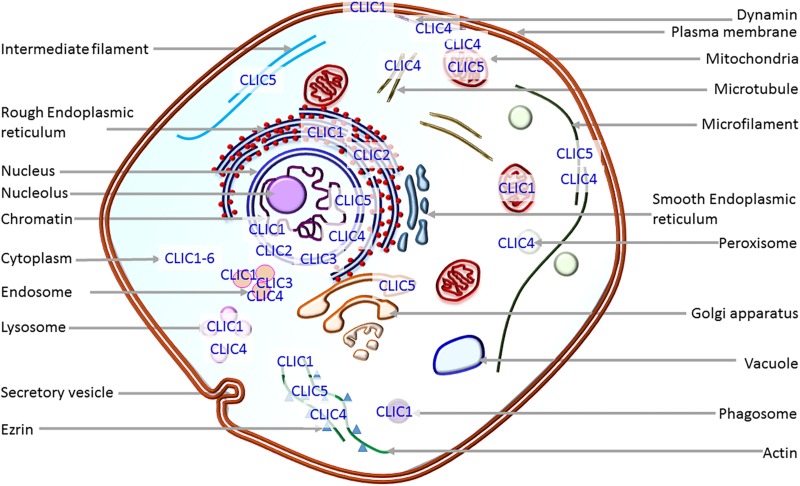
Localization of CLIC proteins. Depiction of the presence of CLICs in intracellular organelles. Except for CLIC1 which goes to the plasma membrane on overexpression, all other CLIC proteins predominantly localize to intracellular organelles.

One of the unique features, which distinguishes CLICs from other ion channels, is their dimorphic existence. They exist in membranous as well as cytosolic or soluble forms (Singh, 2010; Gururaja Rao et al., 2018) and as such, could easily be predicted to play differential physiological roles. In the cytosol, they interact with cytoskeletal filaments and other cytosolic proteins (Figure 1). These interactions with cytoskeletal filaments could be responsible for their functional outcome as well as transportation and translocation (Suginta et al., 2001; Singh et al., 2007; Argenzio et al., 2014; Argenzio and Moolenaar, 2016). Moreover, the cytosolic soluble version of CLIC proteins have recently been shown to possess enzymatic activity in the case of CLIC1 (Al Khamici et al., 2015). CLIC1 has a conserved active site with a glutaredoxin monothiol motif, similar to that of Glutathione S transferases. This site is shown to carry out glutathione-dependent oxidoreductase activity. Hence apart from the widely reported channel activity, these proteins also have a role as enzymes while they are present in their soluble form in the cytoplasm. It is also worth noting that CLICs can be regulated by post-translational modifications, such as phosphorylation (Guo et al., 2018), wherein cyclin-dependent kinase 5 mediated phosphorylation of a serine on CLIC4 increases its stability and regulates apoptosis. This places CLICs in the class of ‘moonlighting proteins’ (Piatigorsky and Wistow, 1989; Jeffery, 1999) where they can perform different, and perhaps, interdependent functions at the membrane and the cytoplasm.

Cytosolic form structure of most of the CLIC proteins is known and is identical to the glutathione S-transferase superfamily (Cromer et al., 2002). They all possess omega-superfold in the C-terminus of the protein (Cromer et al., 2002; Argenzio and Moolenaar, 2016) and cytosolic CLIC1 also shows glutaredoxin-like enzyme activity (Al Khamici et al., 2015). Crystal structure of membrane form is not yet elucidated, but structure-function experiments show that CLICs possess a transmembrane domain in the N-terminus region (Averaimo et al., 2013; Gururaja Rao et al., 2018). The major outstanding question in the field is how a soluble protein unfolds and inserts into the membrane to form a functional ion channel. The key factors involved in this transition are still being investigated (Fanucchi et al., 2008; Stoychev et al., 2009; Legg-E’silva et al., 2012; Argenzio et al., 2014; Peter et al., 2014; Hare et al., 2016). CLIC proteins are known to interact with several cytoskeletal filaments and intracellular proteins that can regulate their function or vice versa. Several members of the CLIC family, CLIC1 to CLIC6, located in various organelles of the cells (Figure 1) are implicated in physiological roles and pathological conditions, such as cancer initiation and progression in multiple studies (Peretti et al., 2014; Flores-Tellez et al., 2015; Hernandez-Fernaud et al., 2017), pulmonary hypertension (Wojciak-Stothard et al., 2014), hearing (Gagnon et al., 2006; Seco et al., 2015), Alzheimer’s disease (Novarino et al., 2004; Milton et al., 2008) and cardiac dysfunction (Takano et al., 2012). CLICs are extensively studied in cancer and tumor growth, and display differential expression and localization in cancer cells during metastasis (Suh and Yuspa, 2005; Peretti et al., 2014). In this review, we have focused on the information available on each member of the CLIC family with respect to their physiological and pathological roles, with emphasis on how they are modified in human tumor scenarios. For the role of VDAC (the other intracellular chloride channel) in cancer, please refer to the recent reviews (Mazure, 2017; Shoshan-Barmatz et al., 2017), and for ion channels in general, please refer (Hille et al., 1999; Jentsch et al., 2004; Amin et al., 2010; Roelfsema et al., 2012; Feske et al., 2015; Prindle et al., 2015; Bates, 2015; Sabirov et al., 2016; Jentsch, 2016; Prevarskaya et al., 2018).

## CLICs in Cardiovascular and Pulmonary Function

The expression of CLIC proteins in the cardiovascular system is heterogeneous. All of the CLICs are found in the majority of cell types, but the precise localization and distribution of CLICs are not yet established in all cell types. CLICs were originally affinity-purified by using IAA-94. IAA-94 is used in several cardiovascular studies and has been shown to impact the myogenic response of cerebral arteries in the presence of potassium ions (Nelson et al., 1997). However, concentrations used (200 μM) rendered non-specific effects and also blocked L-type Ca^2+^ channels (Doughty et al., 1998). These results were not conclusive for the role of any of the CLICs in vascular function. Recently, CLIC2 was found in endothelial cells in blood vessels, but not lymphatic vessels, in non-cancerous tissues, and its expression is significantly lower when compared to endothelial cells of blood vessels in cancerous tissues. Ablation of CLIC2 in human umbilical vein endothelial cells (HUVECs) compromised the integrity of the vessels and allowed human cancer cells to transmigrate through a HUVEC monolayer (Ueno et al., 2019). In addition to CLIC2, CLIC1, and CLIC4 are also known to be present in endothelial cells (Suh et al., 2005b; Money et al., 2007; Tung and Kitajewski, 2010; Wegner et al., 2010), and CLIC5 is present in placental as well as glomerular endothelial cells (Money et al., 2007; Wegner et al., 2010).

The role of CLICs in tubulogenesis and angiogenesis is well established. In *Caenorhabditis elegans*, a CLIC homolog (Exc-4) was shown to localize to the luminal membrane of excretory tubes and is required for tubule formation and its maintenance (Berry et al., 2003). In mammals, CLIC4 is localized to vesicles in HUVECs (Bohman et al., 2005) and large vacuoles in mouse heart endothelial cells (Ulmasov et al., 2009). Removal of CLIC4 in mice revealed stunted vascular development when challenged by oxygen toxicity (Ulmasov et al., 2009). CLIC4 was found to be present in midbody and centrosome of cultured bovine aortic endothelial cells as well as at the cell-cell junction, implicating it in establishing or maintaining cell polarization (Berryman and Goldenring, 2003). In *in vitro* studies, CLIC4 was shown to promote endothelial cell proliferation and regulate endothelial morphogenesis (Tung et al., 2009) possibly by acidification of vacuoles (Ulmasov et al., 2009). In line with worm Exc-4 and mammalian CLIC4, CLIC3 mRNA and protein expression has been showed to be significantly increased in preeclampsia patients (Enquobahrie et al., 2008; Murthi et al., 2012). Preeclampsia is a pregnancy-related condition characterized by endothelial dysfunction in the placenta. To clearly understand the role of CLICs in the vascular system, comprehensive studies need to be performed in null mutant mice.

One of the most significant pathological roles discovered for CLICs was their involvement in pulmonary hypertension (PH). PH is characterized by a loss of vasodilator influences in the pulmonary circulation, which results in pathogenic vasoconstriction, and remodeling of small intrapulmonary arteries, leading to eventual right heart failure (Umar et al., 2012). CLIC4 was found to be highly expressed in the pulmonary vascular endothelium of PH patients (Wojciak-Stothard et al., 2014). Surprisingly, ablation of CLIC4 rendered protection to the null mutant mice and these animals did not develop PH when exposed to hypoxia (Wojciak-Stothard et al., 2014). Later, Arf6 was shown as a novel effector of CLIC4 and proposed as a new therapeutic target in PH (Abdul-Salam et al., 2019). The discovery of the involvement of CLIC4 in PH is highly significant and implicates a role for CLIC4 in lung remodeling. However, it is not clear whether other CLICs can compensate for the loss or overexpression of CLIC4 in PH. For example, patients treated with pirfenidone for idiopathic pulmonary fibrosis presented changes in contrasting expression of CLIC3 and CLIC6 (Kwapiszewska et al., 2018).

In the heart, CLICs are extensively expressed in several types of cells (Gururaja Rao et al., 2018). CLIC1, CLIC4, and CLIC5 were localized in adult cardiomyocytes, and further CLIC4 and CLIC5 were localized to mitochondrial membranes (Ponnalagu et al., 2016a, b). In fact, CLIC5 is the first chloride channel to be identified up to the molecular level in the inner membrane of cardiac mitochondria (Ponnalagu et al., 2016b). The first member of the CLIC family to directly implicate them in cardiac dysfunction was CLIC2. Using exome analysis and deep sequencing of genes on the X-chromosome, a mutation in CLIC2 (c.303C>G, p.H101Q) was discovered, which was found to be associated with X-linked intellectual disability (ID), atrial fibrillation, cardiomegaly, congestive heart failure (CHF), and seizures. CLIC2 is known to interact with ryanodine receptor (RyR) proteins and inhibit its activity. However, the CLIC2 H101Q variant stimulated, rather than inhibited, the action of RyR channels. In blood cells, CLIC1 is known to promote platelet function, promote adhesive functions in platelets as well as endothelial cells, and is critical for vascular repair and angiogenesis (Knowles et al., 2018).

One of the major causes of morbidity and mortality in myocardial infarction is due to ischemia-reperfusion (IR) injury (Hausenloy and Yellon, 2013). Using the known CLIC-blocker, IAA-94, several groups have shown that CLICs could possibly be involved in protecting the heart from IR injury. IAA-94 increased cell death *in vitro* as well as size of myocardial infarction *in vivo* models, respectively. Cyclosporine A, which is known to protect the heart, was rendered ineffective in the presence of IAA-94 (Diaz et al., 1999, 2013; Batthish et al., 2002). We have recently discovered that IAA-94 reduces the calcium retention capacity of mitochondria, and cyclosporine A cannot reverse or prevent the deleterious effect of IAA-94 (Ponnalagu et al., 2019). The absence of CLIC4 also increases mitochondrial ROS, which has been shown to be associated with IR injury as well (Ponnalagu and Singh, 2016; Ponnalagu et al., 2016b). However, these pharmacological studies are to be extended to include genetic models, rapid *in vivo* approaches (Kohut et al., 2016; Patel et al., 2018), and comprehensive studies involving other CLIC proteins to assign a definitive role to CLICs in protecting the heart from IR injury and heart function.

## CLICs in Hearing Impairment

One of the major physiological roles of CLIC5 is implicated in hearing impairment. In a consanguineous Turkish family diagnosed with autosomal recessive non-syndromic hearing impairment (arNSHI), a homozygous nonsense mutation c.96T>A [p.(Cys32Ter)] was observed (Seco et al., 2015) in CLIC5 locus. The mutation in CLIC5 is projected to result in nonsense-mediated mRNA decay, since it creates a premature stop codon [p.(Cys32X)] 54 bp upstream of the 3′-most intron. The initial hearing loss in the group of patients was mild, mainly affecting the mid and high frequencies, but later progressed to severe-to-profound hearing loss. However, the mutations were not the common cause of arNSHI in the Netherland and Spanish patient population. Analysis of expression of CLIC5 in the human fetal inner ear with other adult and fetal-stage human tissues revealed that the expression of CLIC5 in the fetal inner ear was 26-fold higher than in fetal liver in which the expression level was the lowest. These findings mark the importance of CLIC5 in hearing impairment in a specific population.

Sharing the hearing loss and complete vestibular dysfunction, mice with *jitterbug* (*jbg*) mutation also resemble the human hearing impairment phenotype. In wild type mice, CLIC5 is present in stereocilia of both cochlear and vestibular hair cells as well as the apical surface of Kolliker’s organ during cochlear development. In *jbg* mice, CLIC5 is completely absent, accompanied by dysmorphic stereocilia and progressive hair cell degeneration. In younger mice (1–5 months old), auditory-evoked brainstem responses were present in *clic5*^–^*^/^*^–^ mouse but were 40–50 dB higher than wild type mice (Gagnon et al., 2006). However, with the progression of age at around 7 months (∼38 years for humans) *clic5*^–^*^/^*^–^ mouse presented complete deafness due to progressive hair bundle degeneration and a reduced density of spiral ganglion cells (Gagnon et al., 2006). The vestibular hair cells of *jbg* mice also showed a progressive degeneration, but there were no significant changes observed in wild type mice between 5 and 7 months of age (Gagnon et al., 2006). In the crista ampullaris of *jbg* mice, the number of vestibular hair cells was lower than the wild type, whereas hair cells were nearly absent at 12 months (Gagnon et al., 2006). At molecular levels, CLIC5 is shown to work with cytoskeletal elements such as radixin, protein tyrosine phosphatase receptor Q, taperin, and myosin VI. These interactions are essential to stabilize membrane-cytoskeletal attachments at the base of the hair bundle (Salles et al., 2014). Absence of CLIC5 compromises the stability of hair bundles either by disrupting cytoskeletal filaments or by disrupting Cl^–^ transport (Singh et al., 2007) in hair cells, resulting in the progressive loss of integrity of these vital structures.

## CLICs in Neurophysiology

Chloride Intracellular Ion Channel proteins are known to be present in neurons and astrocytes (Berryman and Bretscher, 2000; Novarino et al., 2004; Milton et al., 2008). At the functional level, CLIC1 is characterized for its role in Alzheimer’s disease (AD) where it is present in activated microglia. The expression of CLIC1 is dramatically increased (60%) in the hippocampus of mild/moderate AD patients. In experimental settings, Aβ stimulation of either primary rat microglia or the microglial cell line BV2 enhances expression of the CLIC1 protein and strengthens anion permeability with an increase in conductance mediated by CLIC1 (Novarino et al., 2004; Milton et al., 2008; Paradisi et al., 2008). In patch-clamp, Aβ directly increases the open probability and mean open time of CLIC1 (Milton et al., 2008). Addition of blockers or siRNAs for CLIC1 reduces the neurotoxicity by downregulating the TNFα and ROS. Even in a mouse model of AD, CLIC1 localizes to the plasma membrane of activated microglia. CLIC1 migration to the plasma membrane causes an increase in Cl^–^ permeability across the cell membrane and results in the Aβ-mediated cytotoxic effect. It is still not clear how CLIC1 mediates these cytotoxic effects, but it is a possibility that CLIC1 could interact with other ion channels or form a channel itself.

Apart from CLIC1, CLIC2 is also implicated in neurophysiology. In a large-scale next-generation sequencing of X-chromosome genes, a mutation in CLIC2 was identified on Xq28 in a male with X-linked intellectual disability (XLID) (Witham et al., 2011). There are several non-synonymous SNPs reported for CLIC2 in healthy individuals which do not affect its function. However, p.H101Q lessens the flexibility of the joint loop and reduces the possibility of the large conformational change which is expected to occur when CLIC2 changes from its cytosolic form to membranous form. Further, the H101Q mutation is known to stimulate rather than inhibit the activity of RyR channels (Takano et al., 2012) which could explain seizures in patients with XLID.

In ischemia-induced neuronal apoptosis, expression of CLIC4 was shown to be increased. Furthermore, activated cyclin-dependent kinase 5 (CDK5) is shown to phosphorylate serine 108 of CLIC4 which increases the stability and accumulation of CLIC4 (Guo et al., 2018). In contrast, inhibition or downregulation of CDK5 decreased CLIC4 levels in neurons (Guo et al., 2018); overexpression of CLIC4 caused H_2_O_2_-induced neuronal apoptosis. These findings suggest that overexpression of CLIC4 could be determinantal for cell survival, which could, again, be due to change in Cl^–^ concentration in cellular compartments.

## CLICs in Cancer

One of the major pathophysiological roles assigned to CLICs is in cell growth and apoptosis. Unregulated cell growth can result in tumor development and cancer. Given the ubiquitous expression of CLICs in all cells, they are also found in most tumors. In this section, we have categorized individual CLICs with respect to their roles in different types of cancers and performed analysis with their expression with respect to patient survival in human cancers.

### CLIC1

CLIC1 is overexpressed in several cancers including liver cancer, gall bladder cancer, pancreatic ductal adenocarcinoma, glioma, breast cancer, nasopharyngeal carcinoma, and gastric cancer to name a few (Wulfkuhle et al., 2002; Petrova et al., 2008; Chang et al., 2009; Wang et al., 2009, 2018; Tang et al., 2012; Wei et al., 2015; Jia et al., 2016; Qu et al., 2016). It is known to play a role in cell viability, possibly by modulating mitochondrial function. CLIC1 is a biomarker for ovarian cancers (Ye et al., 2015) holding prognostic value (Yu W. et al., 2018). Along with CLIC4, CLIC1 is a promising biomarker for epithelial ovarian cancer where its expression predicts patient survival (Singha et al., 2018). CLIC1 is shown to regulate the redox-sensitivity of ovarian cancer cells and is predicted to be a potential marker for lymphoblastic leukemia (Qu et al., 2016; Dehghan-Nayeri et al., 2017; Singha et al., 2018). Modulation of CLIC1 expression by micro RNA, hsa-mir-372 predicts the poor prognosis of patients affected with gall bladder cancers (Zhou et al., 2017) and is important for the migration and invasion of gall bladder cancer cells (He et al., 2018). CLIC1, along with CLIC4, contributes to the virus-mediated motility of Merkel cell carcinoma (Stakaityte et al., 2018).

CLIC1 was also shown to be overexpressed in pancreatic cancer tissues. Furthermore, siRNA targeting CLIC1 mRNA with a subsequent decrease in CLIC1 expression lead to a decrease in cell proliferation (Lu et al., 2015). This work ascertains that CLIC1 expression is integral to the development and progression of pancreatic cancer. Jia et al. (2016) showed increased CLIC1 expression in pancreatic ductal adenocarcinoma correlating higher expression with higher histological grade and increased tumor size. Two independent groups have shown that in hepatocellular carcinoma CLIC1 is overexpressed (Song et al., 2010), and, surprisingly, down-regulation of CLIC1 enhanced the proliferative activity and blocked apoptosis (Li et al., 2012). In studies focused on cell cycle, IAA-94, a CLIC blocker (Valenzuela et al., 2000) was shown to arrest cells at the G2-M phase linking CLICs to the cell cycle. While the mechanism of how CLIC1 is involved in the cell cycle is yet to be explored, these studies do place CLIC1 as an important molecule for cell cycle progression.

CLIC1 is not only important for cell cycle progression in cancer cells but also influences cell migration and metastatic invasion. Blocking CLIC proteins with IAA-94 reduced reactive oxygen species (ROS) generation (Ponnalagu and Singh, 2017) and is hypothesized to block migration and invasion in colon cancer and laryngeal cancer cells (Kim et al., 2016). Blocking CLIC1 with RNAi also showed a similar effect proving that CLIC1 indeed is important for metastasis (Wang et al., 2012, 2014). One of the hallmarks of malignancies is rapid and often uncontrolled angiogenesis to provide blood supply to tumor tissue. A consequence of this unregulated angiogenesis is a constant hypoxia-reoxygenation state leading to increased reactive oxygen species (ROS) production, providing a substrate for further undifferentiation. CLIC1 has been shown to be overexpressed in gaster cancer cells where it is associated with increased production of ROS in a hypoxia-reoxygenation induced state. Furthermore, functional inhibition of CLIC1 downregulates ROS production in gastric cancer cells and decreases gastric cancer cell migration and invasion (Zhao et al., 2015). It is intriguing that CLICs have a close association with mitochondria and are involved in the regulation of ROS (Suh et al., 2004; Ponnalagu et al., 2016a, b). In this context, CLIC1 could be predicted to regulate ROS generation in tumor cells. CLIC1 also modulates MAP kinase and AKT signaling to promote gastric cancer (Li et al., 2018), which could implicate CLIC1 in modulating signaling pathways through ROS.

Glioblastoma is an aggressive and common tumor type where CLIC1 is highly expressed (Wang et al., 2012; Setti et al., 2013). In these cells, CLIC1 suppression reduced both proliferation and self-renewal properties. In glioblastoma, CLIC1-mediated channel activity was recorded to distinguish cytosolic vs membranous components (Setti et al., 2013). The currents correlate with the tumor aggressiveness indicating a positive correlation between membrane form of CLIC1 to glioblastoma, and these results indicate that CLIC1 could be translocated to the membranes in tumor environments. Similar to gastric cancer cells, CLIC1 is shown to regulate ROS accumulation and pH changes in human glioblastoma stem cells influencing their proliferation as well as their motility, and therefore could be a crucial therapeutic target (Gritti et al., 2014; Peretti et al., 2018). CLIC1 was one among the nine genes identified in a screen for ion channels strongly modified in solid tumors and vascular malformations, especially in glioblastoma and bladder cancers (Biasiotta et al., 2016). CLIC1 expression is also correlated with the expression of drug resistance protein MRP1, whereas CLIC1 knockdown decreased its expression in human choriocarcinoma cell lines (Wu and Wang, 2017). Surprisingly, extracellular vesicles were reported to transfer CLIC1 from glioblastoma cells to microvascular epithelial cells (Thuringer et al., 2018), perhaps contributing to metastasis in targeting cells.

In addition to IAA94, CLIC1 is shown to be inhibited by metformin, a drug that effectively blocks cancer stem cell viability (Gritti et al., 2014). Metformin was able to block IAA-94-sensitive currents in tumor-derived stem cells but not normal stem cells, thus opening an exciting possibility that CLIC1 could be localized at the membranes only in tumor environments. In an independent study, CLIC1 partially decreased the antineoplastic effects of metformin and upregulation of CLIC1 increased drug sensitivity (Liu et al., 2017); this again proves CLIC1 can be a therapeutic target for various tumors. A recent study also showed that several drugs of biguanidine class, including metformin, act via CLIC1 (Barbieri et al., 2019). Metformin is presented as a wonder drug in several pathological conditions such as diabetes, and if CLIC1 directly interacts with metformin, it could possibly provide the “elusive” mechanism of action for the drug.

**FIGURE 2:**
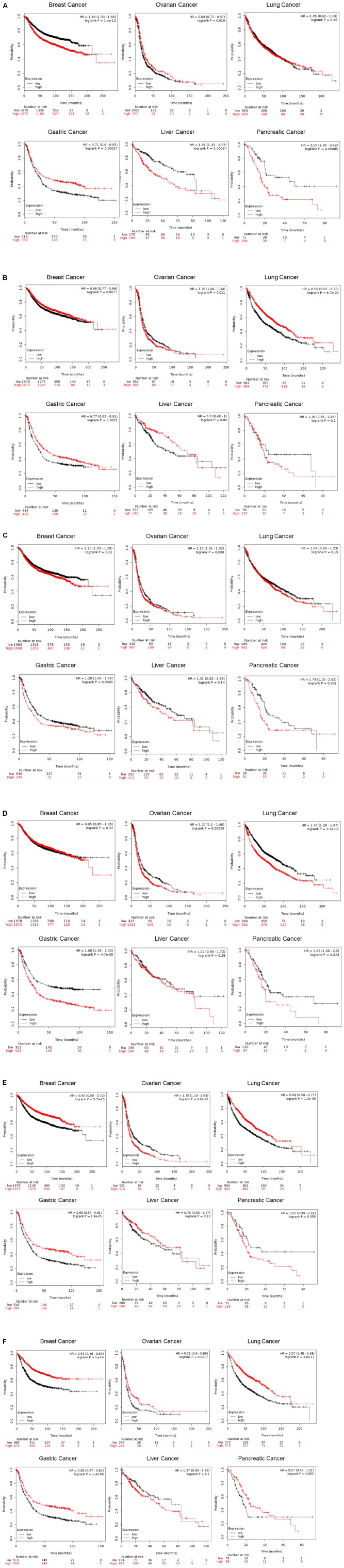
Correlation of CLIC1 expression in various cancers and patient mortality. Kaplan-Meier plot of patients with breast, ovarian, lung, gastric, liver and pancreatic cancers. Comprehensive and updated data were obtained from https://kmplot.com/analysis/ for CLIC1, 2, 3, 4, 5, and 6 and plotted. The K-M plotter assesses the effect of 54k genes on survival during various cancer types. The K-M database includes gene chip and RNA-seq data and the sources for the databases include GEO, EGA, and TCGA. The primary purpose of the tool is a meta-analysis based discovery and validation of survival biomarkers. The red and black line represents high and low expression of CLIC1 **(A**, identification number 208659), CLIC2 **(B**, identification number 213415), CLIC3 **(C**, identification number 219529), CLIC4 **(D**, identification number 201559), CLIC5 **(E**, identification number 213317), and CLIC6 **(F**, identification number 227742), respectively. The number of patients at each time point is given in black (low expression) and red (high expression).

While being the most studied member of the CLIC family in the context of tumors, CLIC1 has the potential to be a diagnostic marker for several cancers as well as being a potential target for therapy. In ovarian cancer, expression of CLIC1 was shown to be directly related to patient survival outcome (Singha et al., 2018; Yu W. et al., 2018). We have performed *in silico* analysis of expression of CLIC proteins and their relation to various cancer related patient mortality and found strong correlations. As shown in Figure 2A, Kaplan-Meier (K-M) (Nagy et al., 2018) analysis of the data collected from several human cancers showing patient survival as a function of the relative mRNA levels (high, red or low, black) indicates CLIC1 high expression correlates to poor patient survival in pancreatic, breast and liver cancers. Low or high CLIC1 expression in the lung or ovarian cancers does not influence patient survival. Surprisingly, gastric cancer patients with higher expression of CLIC1 have a higher rate of survival. These observations from patient samples indicate that not only is CLIC1 important in cancer, but the level of its expression can determine patient survival by specific cancer. This information is helpful in targeting CLIC1 for cancer therapy as well as a prognosis marker. Please refer to Table 1 for a complete list of all six CLIC proteins high/low expression and patient survival chance correlation.

**TABLE 1:** Correlation of CLIC expression with patient mortality in different cancers.

Mortality-CLIC expression correlation	Breast	Ovarian	Lung	Gastric	Liver	Pancreatic
CLIC1	High	Low	Not changed	Low	High	High
CLIC2	Low	High	Low	Low	Low	High
CLIC3	High	High	High	High	High	High
CLIC4	High	High	High	High	High	High
CLIC5	Low	High	Low	Low	Low	High
CLIC6	Low	Low	Low	Low	High	Low

### CLIC2

Understanding of the involvement of CLIC2 in cancer cells or tumorigenesis is limited. CLIC2 is not reported as a cancer gene in the network of cancer genes’ (NCG 6.0) collection of sources of known cancer genes and 273 cancer mutation screenings (Repana et al., 2019). However, recently it was shown that CLIC2 is highly expressed in non-cancerous cells surrounding cancer masses (Ueno et al., 2019). Expression of CLIC2 and other tight junction proteins such as claudins 1 and 5, occludin and ZO-1 were significantly higher in non-cancer cells as compared to human hepatocellular carcinoma. These keys findings implicate CLIC2 in the formation or maintenance of tight junctions which the cancer cells lack. Increasing expression of CLIC2 in cancer or surrounding cells could prevent the invasion of cancer cells, and thus local progression and distant metastasis. In thymic epithelial tumors, expression of CLIC2 along with ABCE1 was higher. CLIC2 was also detected in type B1 and B2 thymomas (Zhao et al., 2018). In human hepatocellular carcinoma and colorectal cancer cells, CLIC2 was shown to be important in forming tight junctions in the cancer vasculature and could be playing a role in preventing metastasis (Ueno et al., 2019). However, while there is a lack of extensive studies on CLIC2 in animal models, it is differentially associated with patient survival in various cancers as shown in K-M plots (Figure 2B). High levels of CLIC2 are correlated with better survival in breast, lung, liver and gastric cancers. Whereas, in pancreatic cancer, there is an inverse relationship with lower survival associated with high expression of CLIC2. Surprisingly high expressions of CLIC2 and CLIC5 have the same outcome on patient mortality at given time points.

### CLIC3

CLIC3 has also been shown to be involved in malignancies. Cancer-associated fibroblasts produce a drastic change in the secretome surrounding cancer cells. CLIC3 was found in the stroma surrounding fibroblasts associated with breast cancer, which is known to be in part secreted by breast cancer-associated fibroblasts (Hernandez-Fernaud et al., 2017). CLIC3 is implicated in promoting cell invasion, and increased CLIC3 levels in the stroma is associated with increased cell invasion capabilities. It has also been demonstrated that CLIC3 is a glutathione-dependent oxidoreductase, and its function has a downstream reduction in transglutaminase-2 binding with its cofactor leading to a decrease in transglutaminase-2 activity, which is essential for physiologic function and regulation of extracellular stroma (Hernandez-Fernaud et al., 2017). Recently, CLIC3 was also proposed to be a prognostic marker for lung cancer (Liu et al., 2019).

In salivary gland mucoepidermoid carcinoma, CLIC3 gene expression was markedly elevated compared to normal tissue (Wang et al., 2015). It was further demonstrated that CLIC3 has a differential methylation profile in tumor samples compared to normal samples and was subsequently correlated with the increase in gene expression to increased protein expression in the same tumor samples. However, mechanistic details of CLIC3 in tumors remain to be studied in detail. Our analysis of CLIC3 as shown by K-M plots in Figure 2C shows how levels of CLIC3 mRNA expression are correlated with patient survival in tumors. Interestingly, high CLIC3 expression correlates with a lower patient survival rate in all six cancers listed in Figure 2.

### CLIC4

CLIC4 is the other well studied CLIC member in tumor biology, along with CLIC1. CLIC4 was originally proposed as a mitochondrial CLIC (mtCLIC) (Fernandez-Salas et al., 2002; Arnould et al., 2003; Suh et al., 2004; Xu et al., 2013) and recently was localized to the outer membrane of mitochondria (Ponnalagu and Singh, 2016; Ponnalagu et al., 2016b). Yuspa and colleagues in 2005 reported for the first time revealed that while CLIC4 expression is reduced in tumor cells, its expression was elevated in stromal cells (Suh et al., 2005a, b; Suh et al., 2007a; Suh et al., 2012). Extensive studies by Yuspa’s group have revealed CLIC4 to have a dual role in tumor environment where its expression is lost early in tumor cells while it is increased in stromal cells. The loss of CLIC4 has also been demonstrated in the tumor environments of squamous tumors of the esophagus (Suh et al., 2007a). Replacing the tumor expression of CLIC4 is shown to repress tumor growth as shown by *in vitro* studies in tumor cell culture as well as orthografts in *in vivo* models (Suh et al., 2012). Mechanistically, CLIC4 is hypothesized to be acting through the TGF-β signaling pathway (Shukla et al., 2009, 2013, 2016; Shukla and Yuspa, 2010). CLIC4 enhances TGF-β responsiveness by dephosphorylating Smad in association with Schnurri-2 (Shukla et al., 2009) and by amplifying the expression of Smad7Δ, a splice variant of Smad (Shukla et al., 2016).

CLIC4 was also shown to be upregulated in stromal cells of breast cancer patients as a response to TGF-β treatment (Ronnov-Jessen et al., 2002). It is anticipated to be involved in the acidification of vacuoles in stromal cells contributing to angiogenesis in the process of endothelial tube formation (Ulmasov et al., 2009). Given the association with TGF-β pathway, CLIC4 could be directly involved in ROS mediated mechanisms for tumor growth (Yao et al., 2009). CLIC4 expression is shown to be elevated in human oral squamous carcinoma compared to normal controls (Xue et al., 2016). This study demonstrated that knockdown of CLIC4 expression by siRNA leads to increased apoptosis, mediated by enhanced ATP and thapsigargin induced calcium release from endoplasmic reticulum calcium stores (Xue et al., 2016). It would be interesting to investigate how endoplasmic reticulum/mitochondrial localization of CLIC4 could possibly be affecting tumor metabolism. CLIC4 is shown to exhibit varied expression and activity in several tumor types including ovarian, colon, bladder cancers, glioma, melanoma and even present in the exosomes ejected from human ovarian cancer cell lines. CLIC4 expression is positively correlated with tumor grade, lymph node metastasis, tumor invasion and poor overall survival in ductal adenocarcinoma of the pancreas (Bae et al., 2004; Dyrskjot et al., 2004; Alonso et al., 2007; Liang et al., 2013; Zou et al., 2016). Curiously, knockdown of CLIC4 in mouse liver cancer cells promotes apoptosis (Yu Q.Y. et al., 2018).

The dual existence of CLIC4 as a membrane and cytosolic protein as well as localization to various intracellular organelles within the cytoplasm makes it a challenging structure to study. CLIC4 is shown to be present in the mitochondria of keratinocytes and cardiomyocytes (Fernandez-Salas et al., 2002; Ponnalagu et al., 2016a, b), and is also shown to regulate mitochondrial function. Additionally, CLIC4 is known to translocate to the nucleus under cellular stress (Suh et al., 2004). It is not clearly established how relevant the nuclear translocation is in cancer and whether this translocation interferes with cell cycle or transcription signaling process. The status of CLIC4 in tumor vs. normal scenarios in terms of a membrane protein acting as a channel or cytoplasmic protein performing other functions for the cell is yet to be elucidated. However, the numerous evidences for altered CLIC4 function and tumor graft studies that show a reduction in tumor formation upon altering CLIC4 expression provide enough basis to study CLIC4 as a target in cancer therapy. Our K-M plot analysis of human tumor mRNA data shows the CLIC4 high expression correlating with poor patient survival in all the six cancers analyzed (Figure 2D). CLIC4 follows the same pattern as CLIC3 and our analysis is in agreement with an earlier report on ovarian cancer (Singha et al., 2018).

### CLIC5 and CLIC6

Amongst CLICs, CLIC 1-4 are the more investigated proteins of the family in relation to cancer. However, there have been reports of changes in the expression of the last two members of the CLIC family. Microarray studies have identified changes in CLIC5 and CLIC6 expression in breast cancer tissues along with CLIC1 and CLIC4 (Ko et al., 2013). Studies also show that CLIC5 undergoes differential methylation in neuroblastoma (Olsson et al., 2016). CLIC5 is the first Cl^–^ channel identified to the molecular level in the inner membrane of mitochondria (Ponnalagu et al., 2016b). CLIC5 is also expressed along with Ezrin and Podocalyxin in hepatocellular carcinoma participating in the invasion and migration of tumors (Flores-Tellez et al., 2015). CLIC5 is overexpressed in childhood acute lymphoblastic leukemia, following the loss of ETV6 (Neveu et al., 2016). Similar to CLIC4, CLIC5 was also reported in mitochondria where it plays a role in modulation of ROS, which could also contribute to tumor signaling. Further in-depth studies regarding the roles in the tumor process are required for CLIC5 and CLIC6.

Recently, hypermethylation islands are discovered in the promoter regions of CLIC6 in a study that identified epigenetic CpG island methylation in adenoid cystic carcinoma (Bell et al., 2011), suggesting CLIC6 being involved in the development of this tumor and may serve as a diagnostic marker. Figure 2E containing K-M plots shows that high CLIC5 expression is related to poor patient survival in ovarian and pancreatic cancers while the low expression correlates with poor patient survival in breast, gastric, liver and lung cancers. On the other hand, CLIC6 high expression indicates better survival in all cancers except liver cancer. This analysis of human data shows that differential expression of CLIC1-6 has varied consequences for patient survival in various human cancers (Figure 2F).

## Concluding Remarks

Given their ubiquitous presence, it is not implausible to imagine that dimorphic CLICs will reveal themselves to be of diagnostic, prognostic, and therapeutic applications for a multitude of human physiological and pathological conditions. As discussed above, CLICs play a key role in cardiovascular, pulmonary, neurological, and auditory function, as well as various malignancies. Levels and expression of CLICs can be used as diagnostic and prognostic markers for these diseases. For example, the direct correlation between CLIC expression and patient mortality illustrated (Figure 2) in this review, present them as promising targets in cancer therapy. In addition, a comprehensive understanding of the exact molecular basis and interactors of the complex signaling networks activated by CLIC proteins in specific disease conditions involved is required, whether they are up or down-regulated.

One of the key organelles where CLICs are known to be localized in is the mitochondria (Ponnalagu and Singh, 2016; Ponnalagu et al., 2016a,b, 2019). CLICs are known to modulate mitochondrial physiology by affecting ROS generation and calcium capacity (Ponnalagu and Singh, 2016; Gururaja Rao et al., 2018). It is possible that CLICs located in mitochondria are the important targets for cell proliferation, and modulating their channel activity will be an effective measure to regulate cell physiology. Mitochondria play a crucial role in cell physiology and survival, and recently several lines of research have drawn links to how mitochondrial energetics, dynamics, and metabolism contributes to diseases (De Vivo and DiMauro, 1990; Wallace, 2005; Balaban, 2006; Schieke and Finkel, 2006; Di Lisa et al., 2007; Brown and O’Rourke, 2010; Kaasik et al., 2010; Nagaraj et al., 2012; Katsetos et al., 2013; Szabo and Zoratti, 2014; Lightowlers et al., 2016; Murphy et al., 2016; Vyas et al., 2016; Gururaja Rao, 2017; Porporato et al., 2018). Hence, it would be intriguing to unravel how the presence of CLICs in mitochondria could contribute to its function in several pathophysiological conditions. Furthermore, the role of CLICs as ion channels or regulators of ion channels needs to be elucidated.

A caveat that needs to be addressed in this context is the functional and spatial correlation of CLICs in pathophysiology. Perhaps the most intriguing layer of complexity with CLICs is that they exist in dimorphic forms where they are present in the soluble cytoplasm form as well as in the ion channel form in various intracellular organelles and the plasma membrane (Singh and Ashley, 2006, 2007; Singh et al., 2007; Singh, 2010; Ponnalagu et al., 2016b; Gururaja Rao et al., 2017, 2018; Ponnalagu and Singh, 2017). Several studies cited in this review have not addressed the outstanding question whether the observed role of the protein in diseases is related to its presence in membranes vs. cytosol. Some of the CLICs, such as CLIC1, are exclusively present in small vesicles, such as lysosomes, where they could be playing a role in acidification (Jiang et al., 2012; Salao et al., 2016); however, the same channel when present in the nucleus could be involved in the regulation of cell cycle (Qian et al., 1999; Domingo-Fernandez et al., 2017). Similarly, CLIC2 interacts directly with ryanodine receptors and modulates their activity but does not conduct any ions by themselves (Board et al., 2004), but a missense mutation in CLIC2 causes intellectual disability and cardiomegaly (Witham et al., 2011; Takano et al., 2012). CLIC4 is present in mitochondrial membranes (Fernandez-Salas et al., 2002; Zhong et al., 2012; Ponnalagu et al., 2016b) where they could play functional channels, but in the cytosol, they interact with dynamin I, and 14-3-3-γ (Duncan et al., 1997; Suginta et al., 2001; Ashley, 2003). CLIC5 is best described for its interaction with cytoskeletal filaments and specifically in hair cells where they play a role in hearing (Berryman and Bretscher, 2000; Shanks et al., 2002; Gagnon et al., 2006; Wegner et al., 2010; Salles et al., 2014; Flores-Tellez et al., 2015). In cardiomyocytes, CLIC5 is present in the inner membrane of mitochondria where it modulates reactive oxygen species (Ponnalagu et al., 2016a, b). Hence, future studies need to focus on these details pertaining to the relation between their localization and function, which would be crucial in completely understanding the scope of CLICs as therapeutic targets. So far, there is no data available to attribute an exclusive role of CLIC protein or CLIC-related ion conductions in pathophysiological functions.

One of the prominent channels, BK_Ca_, is present in the plasma membrane of excitable cells where they play a role in cellular excitability, but the same channel is present in mitochondria of adult cardiomyocytes (Singh et al., 2012b, 2013; Toro et al., 2014). In adult cardiomyocytes, BK_Ca_ results in cardioprotection from ischemia-reperfusion injury. The major reason attributed to this differential distribution of BK_Ca_ is splice variation (Shanks et al., 2002; Friedli et al., 2003; Wegner et al., 2010; Shukla et al., 2016), but the role of these variations in differential localization and function is not yet deciphered. Here, we postulate that splice variation of CLICs (Shanks et al., 2002), their differential localization, and possible ion channel formation in response to various stimuli, such as lower pH or redox, could result in their multiple physiological roles. Our hypothesis is partially substantiated by recent studies showing the splice variation of CLICs (Shanks et al., 2002; Friedli et al., 2003; Seco et al., 2015), their regulation by pH (Warton et al., 2002; Goodchild et al., 2010; Legg-E’silva et al., 2012; Gurski et al., 2015; Zhao et al., 2015; Hare et al., 2016; Peretti et al., 2018) and redox (Littler et al., 2004, 2010; Singh and Ashley, 2006, 2007; Milton et al., 2008; Goodchild et al., 2009; Averaimo et al., 2010; Valenzuela et al., 2013; Al Khamici et al., 2015, 2016), their formation of multi-protein complexes (Berryman and Bretscher, 2000; Suginta et al., 2001; Berryman and Goldenring, 2003; Bohman et al., 2005; Singh et al., 2007; Suh et al., 2007a, b; Ponsioen et al., 2009; Shukla et al., 2013; Patel et al., 2015; Tavasoli et al., 2016; Guo et al., 2018; Abdul-Salam et al., 2019) and their localization to specific membranes (lipid rafts) (Duncan et al., 1997; Ashley, 2003) and cellular organelles (Fernandez-Salas et al., 2002; Arnould et al., 2003; Suh et al., 2004; Chalothorn et al., 2009; Ulmasov et al., 2009; Gomes et al., 2011; Zhong et al., 2012; Ponnalagu et al., 2016b; Tang et al., 2017).

Another major cause for concern in targeting CLIC proteins in a single pathological condition is the ubiquitous nature of the proteins and their involvement in function and regulation of the normal physiological state in cells of varied etiology. Disease hallmarks commonly depend on dysregulation of several channels and associated proteins, and unlike plasma membrane counterparts, intracellular channels generally initiate secondary signaling complexes triggered by cytosolic or extracellular components. Recent advances in the CLICs field has generated the availability of genetic models (Ponnalagu et al., 2016a, b) and innovative techniques (Singh et al., 2009, 2012a; Lee et al., 2016; Gururaja Rao, 2017; Lam et al., 2018) to study CLICs; this will enable the researchers to better assign their roles in pathophysiology including cancer, hearing impairment, AD and vascular dysfunction with respect to individual CLICs in future. This will also shed light on the role of chloride ion in cell physiology, and possibly bring forward novel and unconventional strategies for effective treatment of CLIC-associated diseases.

## Author Contributions

SG and HS analyzed the CLIC data for cancers. SG, NP, and HS collected the information and wrote the manuscript.

## Conflict of Interest

The authors declare that the research was conducted in the absence of any commercial or financial relationships that could be construed as a potential conflict of interest.
